# Factors associated with postoperative complications after orthognathic surgery – a National Swedish register-based cohort study

**DOI:** 10.1007/s10006-025-01493-6

**Published:** 2025-12-04

**Authors:** Carina Pekkari, Carina Kruger Weiner, Adrian Salinas Fredricson, Bodil Lund, Agneta Marcusson, Aron Naimi-Akbar

**Affiliations:** 1 Department of Dental Medicine, Division of Oral Diagnostics and Rehabilitation, Karolinska Institutet, Huddinge, Sweden; 2 Department of Oral and Maxillofacial Surgery, Eastmaninstitutet, Folktandvården Stockholms AB, Stockholm, Sweden; 3 Department of Oral and Maxillofacial Surgery, Gävle Hospital, Folktandvården, Region Gävleborg Gävle, Sweden; 4 Medical Unit for Reconstructive Plastic- and Craniofacial Surgery, Karolinska University Hospital, Stockholm, Sweden; 5 Maxillofacial Unit in Linköping, and Biomedical and Clinical Sciences, Linköping University, Linköping, Sweden; 6 Health Technology Assessment-Odontology (HTA-O), Faculty of Odontology, Malmö University, Malmö, Sweden

**Keywords:** Orthognathic surgery, Postoperative complications, Infections, Jaw fixation techniques, Reoperation, Hypesthesia

## Abstract

**Purpose:**

The aim of the study was to identify patient-specific and/or surgery-specific factors that may predict complications following orthognathic surgery, using data from the Swedish National Register of Orthognathic Surgery (NROK).

**Methods:**

In this retrospective register-based cohort study, data from NROK (2017–2020) was analyzed to identify risk factors for complications following single-jaw maxilla surgery, mandibular surgery, and bimaxillary surgery. Outcome data on postoperative infection (POI), removal of osteosynthesis material, re-operation, and persistent neurosensory disturbance (NSD) were collected 12-months postoperatively.

**Results:**

Among the 428 patients included in the study, 100 had single-jaw maxilla surgery, 130 had mandible surgery, and 198 had bimaxillary surgery. The site of surgery showed to be the main factor linked to postoperative complications after orthognathic surgery. Mandibular interventions and bimaxillary surgery increased the risk of POI, removal of osteosynthesis material and NSD more than three (3) times compared to maxillary surgery. For re-operation, there was no significant difference between the groups. Other factors associated with increased risk were overweight, age over forty, smoking, and no postoperative antibiotics.

**Conclusion:**

Mandibular and bimaxillary procedures are associated with increased postoperative complication rates. Age, BMI, smoking, and antibiotic use should be considered in preoperative planning and postoperative care.

**Supplementary Information:**

The online version contains supplementary material available at 10.1007/s10006-025-01493-6.

## Introduction

Since the 1960s, Sweden has used a system of national registries, managed by various authorities and organizations, to facilitate administration, planning, and research within various sectors of society. In 2017, the National Register for Orthognathic Surgery (NROK) was launched to systematically register orthognathic surgical correction of dentofacial anomalies and growth disorders.

In Sweden, about 900–1000 orthognathic operations are performed annually for correction of skeletal malocclusions [1]. The most common orthognathic surgical procedures performed for correction are Le Fort 1 osteotomy (LFI), bilateral sagittal split osteotomy (BSSO), intraoral vertical ramus osteotomy (IVRO), and genioplasty. All these procedures are performed using an intraoral approach. As the oral mucosa is an environment of normal bacterial flora, with multiple potential pathogens, incisions in this area pose a risk of surgical site infection regardless of rigorous antiseptic techniques [2]. Therefore, orthognathic surgery with intraoral approach is designated as clean-contaminated surgery with an expected infection rate of 10–15% [3]. The downside with the surgery is the risk of intraoperative and postoperative complications. Previously published studies on complications after orthognathic surgery [4–12] consist of data from single clinics; however, this study is new of its kind as it is based on data from a national orthognathic patient register, the NROK register.

The purpose of the study was to use data from NROK to identify factors that could predict postoperative complications after orthognathic surgery, patient-specific factors alone or in conjunction with surgery-specific factors. This knowledge may enhance surgical planning and help reduce complication rates both at an individual and population level.

## Methods

### Study design and registries

This register-based retrospective cohort study consists of gathered data from two nationwide Swedish registries:

- The National Register for Orthognathic surgery (NROK), introduced in 2017, was used to collect data that defined the study population, the main outcome variables, and covariates. In March 2024, NROK had a 78.3% (18 of 23) coverage of all oral and maxillofacial surgery clinics in Sweden performing orthognathic surgery. NROK is administered by Center of Registers Västra Götaland [1, 13].
- The Longitudinal Integrated Database for Health Insurance and Labour Market Studies (LISA), introduced in 1990, was used to collect sociodemographic covariates of interest. Statistics Sweden (SCB) administers the register [14].

Study subjects were collected from NROK from the start of the Register in 2017 to 2020. Complementary data were collected from LISA between 2017 and 2019.

### Study population and data collection

After ethical approval, an application for microdata extracts were sent to the register holders. Data from NROK, including patients’ Personal Identity Number (PIN), a unique number given to all Swedish citizens [15], was sent to SCB. SCB collected the requested data from LISA. The patients were de-identified and given anonymized identification numbers.

Requested data from NROK and SCB were sent to the research group with current identification numbers. Links between the registries were performed using the specific identification numbers given.

Inclusion criteria were patients registered in NROK between 2017 and 2020, operated with orthognathic surgery in one or both jaws, and with a registered follow-up visit 12-months postoperatively. Surgery types included were LFI, segmented LFI, surgically assisted rapid maxillary expansion (SARME), BSSO, IVRO, extraoral vertical ramus osteotomy (EVRO), segmental osteotomy of the mandible, and genioplasty.

Excluded were orthognathic surgical procedures registered in NROK before 2017, patients with no follow-up visit12-months postoperatively, and patients with LFII, LFIII, maxillary- and mandibular osteotomies with distraction.

### Variables

The study period was from the date of surgery to the date of registered follow-up visit 12-months postoperatively in NROK for each patient in the register between 2017 and 2020. Primary outcome variables were postoperative infection in need of antibiotic treatment (yes or no), removal of osteosynthesis material (yes or no), re-operation (yes or no), and persistent neurosensory disturbance 12-months postoperatively (yes or no).

The surgical procedures were divided into three groups: single-jaw maxilla (LFI, segmented LFI, SARME); single-jaw mandible (BSSO, IVRO, segmental osteotomy of the mandible, and genioplasty); and bimaxillary surgery.

The covariates registered were divided into patient-specific and surgery-specific variables. The patient-specific variables (Table 1) included sex, age (a categorical ordinal variable divided into five groups: 15–19, 20–24, 25–29, 30–39, and 40–55), body mass index (BMI), general disease (yes or no), smoking, education level, and living area. Eurostat’s Degree of Urbanization (DEGURBA), 2021 revised definition, was used to divide Sweden’s 290 municipalities into three subgroups: cities (≥ 50 000 inhib), towns and suburbs (≥ 5 000 inhib) and rural areas (< 5 000 inhib) [16, 17]. The surgery-specific variables (Table 2) consisted of operation time (hr.), antibiotic perioperative and postoperative (yes or no), type of osteosynthesis material, and perioperative bleeding volume (ml).

**Table 1:** Patient-specific characteristic of the study population

	Total study population	Single jaw - maxilla	Single jaw - mandible	Bimaxillary surgery
	*n* = 428	*n* = 100	*n* = 130	*n* = 198
**Sex**				
Female (ref)	241 (56%)	52 (52%)	85 (65%)	104 (53%)
Male	187 (44%)	48 (48%)	45 (35%)	94 (47%)
**Age**				
16–19 (ref)	90 (21%)	19 (19%)	30 (23%)	41 (21%)
20–24	222 (52%)	48 (48%)	62 (48%)	112 (57%)
25–29	54 (13%)	16 (16%)	15 (12%)	23 (12%)
30–39	41 (9.6%)	15 (15%)	12 (9.2%)	14 (7.1%)
40–75	21 (4.9%)	2 (2.0%)	11 (8.5%)	8 (4.0%)
**BMI**				
< 18.5	15 (3.6%)	2 (2.0%)	7 (5.5%)	6 (3.0%)
18.5–25.0 (ref)	245 (57%)	59 (59%)	79 (61%)	107 (54%)
> 25.0–30.0	111 (26%)	29 (29%)	24 (18%)	58 (29%)
> 30.0	41 (9.7%)	8 (8.0%)	12 (9.2%)	21 (11%)
Data unavailable	16 (3.7%)	2 (2.0%)	8 (6.2%)	6 (3.0%)
**General disease**	57 (13%)	14 (14%)	16 (12%)	27 (14%)
**Smoking**				
Never been a smoker (ref)	394 (92%)	91 (91%)	119 (92%)	184 (93%)
Stopped > 3 months before surgery	12 (2.8%)	4 (4.0%)	4 (3.1%)	4 (2.0%)
Stopped ≤ 3 months before surgery	11 (2.6%)	5 (5.0%)	2 (1.5%)	4 (2.0%)
Active smoker	11 (2.6%)	0	5 (3.8%)	6 (3.0%)
**Educational level**				
Primary & secondary school (≤ 9 year.)	99 (23%)	24 (24%)	32 (25%)	43 (22%)
Upper secondary school (10–12 year.)	214 (50%)	41 (41%)	63 (48%)	110 (56%)
Higher education (ref)	113 (26%)	34 (34%)	35 (27%)	44 (22%)
Data unavailable	2 (0.5%)	1 (1.0%)	0	1 (0.5%)
**Degree of urbanization**				
Cities (≥ 50 000 inhib)	239 (56%)	51 (51%)	81 (62%)	107 (54%)
Towns & suburbs (≥ 5 000 inhib)	101 (24%)	25 (25%)	28 (22%)	48 (24%)
Rural areas (< 5 000 inhib) (ref)	88 (20%)	24 (24%)	21 (16%)	43 (22%)

Pearson’s Chi-squared Test

Pearson’s Chi-squared Test

**Table 2:** Surgery-specific characteristic of the study population

	Total study population	Single jaw - maxilla	Single jaw - mandible	Bimaxillary surgery
	*n* = 428	*n* = 100	*n* = 130	*n* = 198
**Operation time**				
<2 h	114 (27%)	31 (31%)	65 (50%)	18 (9.1%)
2–4 h	117 (27%)	34 (34%)	39 (30%)	44 (22%)
> 4–9 h (ref)	142 (33%)	28 (28%)	16 (12%)	98 (49%)
Data unavailable	55 (13%)	7 (7.0%)	10 (7.7%)	38 (19%)
**Antibiotic perioperative**	425 (99%)	100 (100%)	128 (98%)	197 (99%)
**Antibiotic postoperative**	192 (45%)	41 (41%)	55 (42%)	96 (48%)
**Osteosynthesis material**				
Osteosynthesis plates (ref)	311 (73%)	89 (89%)	89 (68%)	133 (67%)
Osteosynthesis screws	72 (17%)	0	30 (23%)	42 (21%)
Osteosynthesis plate and screws	23 (5.4%)	0	6 (4.6%)	17 (8.6%)
Others (suture expander)	11 (2.6%)	11 (11%)	0	0
Data unavailable	11 (2.6%)	0	5 (3.8%)	6 (3.0%)
**Bleeding perioperative**				
< 200 ml (ref)	164 (38%)	49 (49%)	84 (65%)	31 (16%)
200–500 ml	209 (49%)	44 (44%)	36 (28%)	129 (65%)
> 500 ml	26 (6.1%)	1 (1.0%)	2 (1.5%)	23 (12%)
Data unavailable	29 (6.8%)	6 (6.0%)	8 (6.2%)	15 (7.6%)

### Statistical analyses

The association between the main exposure variables single-jaw maxilla (SJmax), single-jaw mandible (SJmand), and bimaxillary surgery and the outcome variables were analyzed using Pearson’s chi-square test and binary logistic regression analysis. Odds ratios were calculated with a confidence interval of 95%. Statistical significance was defined as a p-value less than 0.05 (< 0.05) for both statistical tests. The main exposure variables were adjusted for patient-specific variables (Table 1) and surgery-specific variables (Table 2). Patient-specific variables were sex, age, BMI, general disease, smokers, educational level, and DEGURBA. Educational level and DEGURBA were categorized into three groups. Educational level: primary and secondary school (≤ 9 year.), upper secondary school (10–12 year.), higher education. DEGURBA: cities (≥ 50 000 inhib), towns and suburbs (≥ 5 000 inhib), rural areas (< 5 000 inhib).BMI and smokers were categorized into four groups. BMI: <18.5, 18.5–25.0, > 25.0–30.0, > 30.0. Smokers: never been a smoker, stopped > 3 months before surgery, stopped ≤ 3 months before surgery, active smoker.

Surgery-specific variables were operation time, perioperative antibiotic, postoperative antibiotic, osteosynthesis material, and perioperative bleeding. Operation time and perioperative bleeding were categorized into three groups. Operation time: <2 h, 2–4 h, > 4–9 h. Perioperative bleeding: <200 ml, 200–500 ml, > 500 ml. Osteosynthesis material was categorized into four groups; osteosynthesis plates, osteosynthesis screws, osteosynthesis plate and screws, others (suture expander).

Missing values for the covariates BMI, educational level, operation time, osteosynthesis material, and perioperative bleeding were assumed to be Missing At Random (MAR). Multiple imputation by Chained Equations (MICE) was used to impute the missing data (20 imputations) [18, 19]. All statistical analyses were conducted using Stata SE 17.0 (StataCorp LLC, College Station, TX, USA).

## Results

**Fig. 1:**
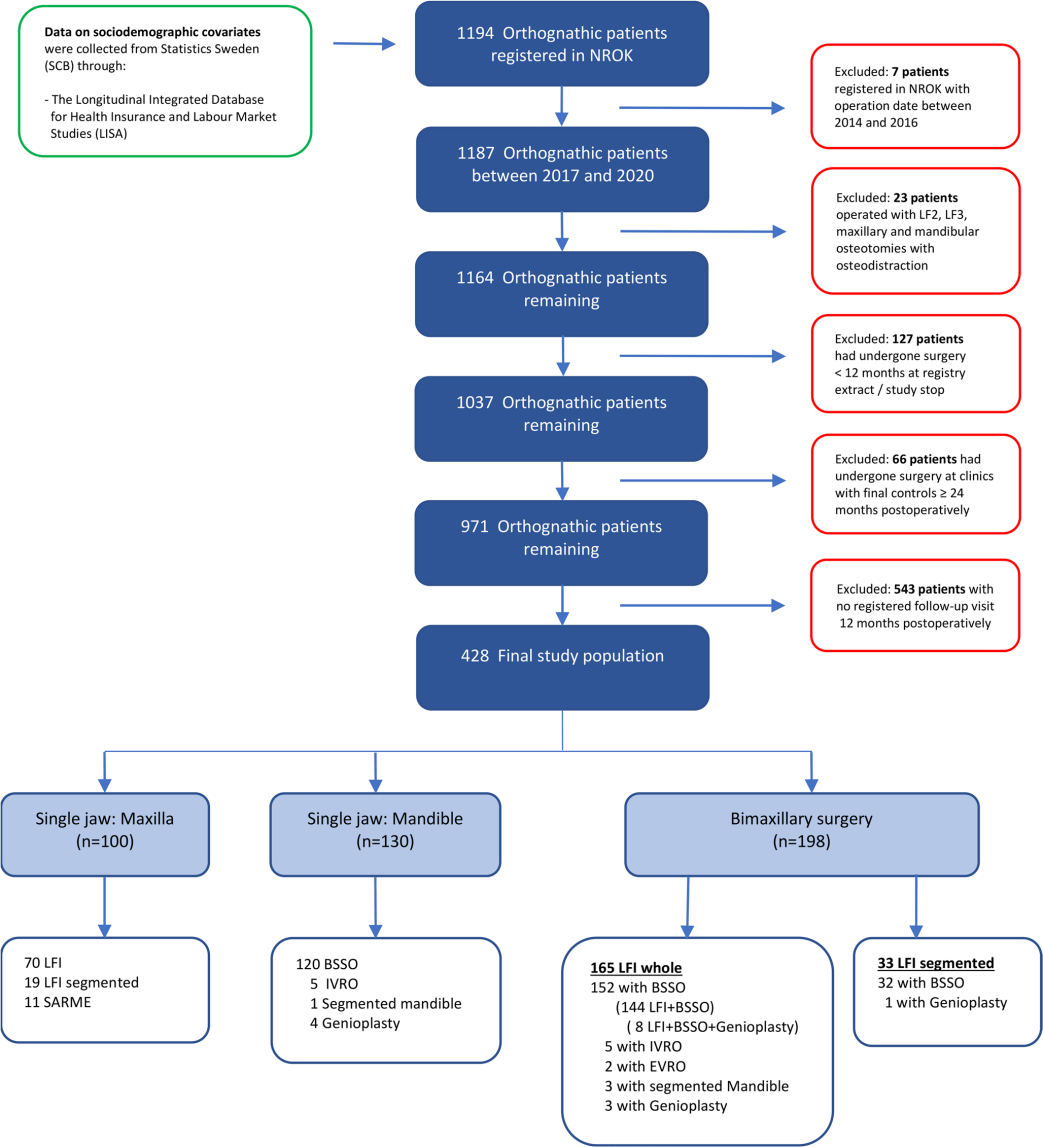
Illustrates the flowchart of the study population. Total population with inclusion and exclusion in different steps

Figure 1 presents the number of eligible patients at each stage of the recruitment. Between 2017 and 2020, 1194 patients were registered in NROK. Seven patients had a date of surgery before 2017, and 23 patients were registered with surgical procedures not included in this study. 127 patients had undergone surgery < 12 months at register extract/study stop, and 66 patients had undergone surgery at clinics with routine of final examinations ≥ 24 months postoperatively. Of the 971 remaining patients, 543 (55.9%) registration in NROK of the last follow-up visit at 12 months postoperative were missing.

Inclusion criteria were met by 428 patients: 100 patients were treated with SJmax, 130 patients with SJmand, and 198 patients with bimaxillary surgery.

Tables 1 and 2 list descriptive data for the study population. In brief, the mean age of the study population was 24 years with a slight female predominance (57%). Most of the patients were healthy with no current or previous smoking habit. Perioperative bleeding of more than 500 ml was rare but reported for 26 patients. For 143 (33%) of the patients, the operation exceeded 4 h.

The proportion of dropouts was analyzed. The total dropout proportion was 55.9% (543 patients of 971). The dropout in the SJmax group was 60%, in the SJmand group 53.6% and in the bimaxillary group 55.1%. There were no major differences in dropouts between the three study groups.

Descriptive data on the background variables for the study population (428 patients) and for the excluded group (543 patients) are presented in their entirety in Supplementary Tables 1 and 2.

When comparing the study population and the excluded group, it is noted that both groups have a relatively similar distribution of background variables.

Missing values for the covariates BMI, educational level, operation time, osteosynthesis material, and perioperative bleeding were assumed to be missing at random and adjusted by multiple imputation (20 imputations) [18, 19]. Table 3 lists the descriptive and missing data for the outcomes postoperative infection (POI), removal of osteosynthesis material, re-operation, and persistent neurosensory disturbance (NSD). Table 4 lists the associations between the main exposure groups and the outcomes. Risk of POI was significantly increased after SJmand (OR 3.103, *p* = 0.018) and bimaxillary surgery (OR 3.529, *p* = 0.008) compared to SJmax for both crude and adjusted data for patient-specific and surgery-specific covariates. BMI < 25.5–30.0 (OR 2.319, *p* = 0.011), living in a larger city ≥ 50,000 inhabitants (OR 2.484, *p* = 0.025), and no use of postoperative antibiotics (OR 2.084, *p* = 0.014) significantly increased the risk of POI.

**Table 3:** Descriptive data of the outcomes

	Total study population	Single jaw - maxilla	Single jaw - mandible	Bimaxillary surgery
	*n* = 428	*n* = 100	*n* = 130	*n* = 198
**Postoperative infection**				
Yes	74 (17.3%)	7 (7.0%)	26 (20%)	41 (20.7%)
No	346 (80.8%)	89 (89%)	104 (80%)	153 (77.3%)
Data unavailable	8 (1.9%)	4 (4.0%)	0	4 (2.0%)
**Removal of osteosynthesis material**				
Yes	56 (13.1%)	5 (5.0%)	19 (14.6%)	32 (16.2%)
No	360 (84.1%)	89 (89%)	110 (84.6%)	161 (81.3%)
Data unavailable	12 (2.8%)	6 (6.0%)	1 (0.8%)	5 (2.5%)
**Re-operation**				
Yes	32 (7.5%)	8 (8.0%)	6 (4.6%)	18 (9.1%)
No	396 (92.5%)	92 (92%)	124 (95.4%)	180 (90.9%)
Data unavailable	0	0	0	0
**Remaining neurosensory disturbance**				
Yes	141 (32.9%)	15 (15%)	46 (35.4%)	80 (40.4%)
No	264 (61.7%)	77 (77%)	81 (62.3%)	106 (53.5%)
Data unavailable	23 (5.4%)	8 (8.0%)	3 (2.3%)	12 (6.1%)

Pearson’s Chi-squared Test

**Table 4:** Postoperative complication rate after orthognathic surgery

		Crude data			Adjusted data for patient-specific covariates			Adjusted data for surgery-specific covariates			Adjusted data for all covariates		
		OR	*p*-value	95% CI	OR	*p*-value	95% CI	OR	*p*-value	95% CI	OR	*p*-value	95% CI
	Single-jaw maxilla (ref)	1		(ref)	1		(ref)	1		(ref)	1		(ref)
**Postoperative infection**	Single-jaw mandible	3.179	0.010*	1.317–7.673	3.393	0.010*	1.344–8.568	3.093	0.014*	1.257–7.612	3.103	0.018*	1.212–7.947
	Bimaxillary surgery	3.407	0.004*	1.466–7.916	3.408	0.006*	1.426–8.147	3.394	0.007*	1.387–8.302	3.529	0.008*	1.393–8.943
**Removal of osteosynthesis material**	Single-jaw mandible	3.075	0.032*	1.104–8.560	2.816	0.056	0.975–8.131	3.032	0.037*	1.068–8.612	2.765	0.066	0.935–8.172
	Bimaxillary surgery	3.538	0.011*	1.331–9.402	3.286	0.020*	1.209–8.933	3.341	0.019*	1.217–9.176	3.191	0.028*	1.133–8.986
**Re-operation**	Single-jaw mandible	0.556	0.293	0.187–1.659	0.387	0.119	0.117–1.278	0.506	0.230	0.166–1.540	0.352	0.094	0.104–1.194
	Bimaxillary surgery	1.150	0.753	0.482–2.744	0.843	0.716	0.336–2.117	1.290	0.589	0.512–3.250	1.016	0.975	0.380–2.720
**Remaining neurosensory disturbance**	Single-jaw mandible	2.915	0.002*	1.505–5.646	2.558	0.008*	1.283–5.102	2.898	0.002*	1.467–5.725	2.489	0.012*	1.217–5.092
	Bimaxillary surgery	3.874	< 0.001*	2.074–7.236	3.988	< 0.001*	2.084–7.632	3.827	< 0.001*	1.951–7.505	3.777	< 0.001*	1.875–7.610

^*^ Binary logistic regression, statistical significance *p* < 0.05

For removal of osteosynthesis material, when compared with SJmax, there was a significantly increased risk for both SJmand (OR 3.075, *p* = 0.032) and bimaxillary surgery (OR 3.538, *p* = 0.011) in the crude data, which remained for bimaxillary surgery (OR 3.191, *p* = 0.028) after adjustment. For re-operation, there was no significant difference between the three study groups. Older patients, 40–75 years (OR 5.315, *p* = 0.044), and smoking (OR 6.001, *p* = 0.024) increased the risk of re-operation.

Risk of NSD was significantly greater after SJmand (OR 2.489, *p* = 0.012) and bimaxillary surgery (OR 3.777, *p* ≤ 0.001) compared to SJmax, both for crude and adjusted data. Female gender (OR 1.863, *p* = 0.009) and no postoperative antibiotics (OR 1.855, *p* = 0.013) significantly increased the risk of NSD. In subgroup analyses of SJmand and bimaxillary surgery, no increased risk or significant differences were found for POI, removal of osteosynthesis material, and NSD between the groups. All analyses of outcome variables and covariates are available in Supplementary Tables 3–10.

## Discussion

The aim of this study was to investigate potential risk factors, patient- and/or surgery-specific, for complications after orthognathic surgery based on data from NROK, a Swedish national patient register for orthognathic surgery. The results of the study showed that site of surgery, specifically interventions in the lower jaw and bimaxillary surgery, was clearly linked to postoperative complications after orthognathic surgery. Mandibular and bimaxillary procedures seems to increase the risk more than threefold for POI, removal of osteosynthesis material, and NSD, compared to maxillary surgery. For re-operation, there was no significant difference between the study groups. Other factors associated with the risk of postoperative complications were overweight, age over 40, smoking, and no postoperative antibiotics. Subgroup analysis of mandibular and bimaxillary surgery did not demonstrate any significant differences between the groups for the four postoperative complications reported.

The dropout in the study signals the phenomenon of missing registration of the final examination 12-months postoperatively. Another factor that may have affected the dropout is the geographic mobility often characteristic for young people. It can be speculated that at the time of the final follow-up, not all operated patients were available for follow-up, as they could have moved from the region for work, educational purposes, or other reasons. In addition, a global COVID-19 pandemic broke out during the study period, with the possible consequence that not all patients were available for final follow-up. The potential confounding factors were dealt with using regression models. The main exposures appeared robust over the different models, yet unknown confounding factors may also be present in the material. One possible confounding factor could be the experience of the surgeons, which was not registered and is known to influence the outcome [20]. Selection bias might, however, be present, as the high overall dropout rate introduces a potential risk of bias in the results. A dropout analysis showed that the study population (428 patients) and the excluded group (543 patients) had a relatively similar distribution of background variables. This reduces the likelihood of a major impact on the outcome, suggesting that the results—despite a dropout rate of 55.9%—can be considered reasonably reliable.

Regarding information bias, one can consider how the data were measured and recorded in the different clinics. However, the definition and interpretation of the different parameters collected in NROK are defined in the online user manual for the register, which reduces information bias.

In total, the incidence of POI was 17%: 7% after SJmax, 20% after SJmand, and 21% after bimaxillary surgery. Other studies report an incidence of POI between 1% and 33% [5, 21–23]. An explanation for the fact that the incidence of POI was higher after SJmand and bimaxillary surgery could be that there is less vascularization in the mandible compared to the maxilla and therefore a vulnerability and an increase in the susceptibility to infection [24]. Another suggested explanation is that bacteria-rich saliva and food debris more easily accumulate along surgical incisions in the lower jaw, forming potential sources of postoperative surgical site infection (SSI) [2, 6, 25]. The risk of POI also increased significantly for patients with BMI < 25.5–30.0, as well as for underweight patients (BMI < 18.5), although this was not statistically significant for the underweight patients. Overweight is well-known to increase the risk of impaired wound healing and higher risk of SSI [26]. The risk for underweight people may be because the immune system is compromised due to nutritional deficiencies, which may affect wound healing and increase the risk of POI. Patients not receiving postoperative antibiotics had twice the risk of developing POI compared to those who received antibiotics. Davis et al. found that 25% of patients without postoperative antibiotics had SSI and only 4% of patients with postoperative antibiotic had SSI. BSSO was involved in 71% of the infections [21]. 

A systematic review by Naimi-Akbar et al., 2018, reported scientific uncertainties regarding the preferred antibiotic substance and optimal interval for prophylaxis in connection with orthognathic surgery [27]. 

Another systematic review by Blatt et al., 2019, showed that prolonged antibiotic regime for orthognathic surgery, may reduce the risk for surgical site infection, but there is a lack of evidence for the effects of short- vs. long-term therapy [28]. 

Presently, existing evidence has not shown that prolonged antibiotic prophylaxis beyond the day of surgery would provide a significant additional anti-infective effect against postoperative infection, but the risk of serious postoperative side effects and the development of antimicrobial resistance increases with prolonged prophylaxis [29]. Living in a larger city (≥ 50,000 inhabitants) was associated with higher risk (OR 2.484) of infection than those living in sparsely populated areas. One possible explanation could be that in more densely populated areas there is a higher risk of exposure to infection than in sparsely populated areas. Another possible explanation is that people living in sparsely populated areas have poorer access to healthcare and opportunity to seek care for postoperative infections, which would lead to underreporting of POI in sparsely populated areas. There are no previously published studies that explicitly address the difference in postoperative infections after orthognathic surgery between densely populated and sparsely populated areas.

Removal of osteosynthesis material was performed in 56 patients (13%) with significantly higher risk after SJmand and bimaxillary surgery than after SJmax. Other studies reported plate removal after orthognathic surgery from 2.8% to 27.5% [11, 23, 25, 30–35]. In previous published studies, smoking [22, 32], increasing age [31, 33], operations performed in the mandible [32], and length of operation [33] are described as risk factors for plate removal. A total of 32 patients underwent re-operation (7.5%). No significant difference was noted between the groups. For smokers and patients 40–75 years of age, the risk of re-operation increased significantly.

Smoking affects the body both at a molecular and cellular level, including peripheral vasoconstriction and tissue hypoxia, which can lead to impaired wound healing and an increased risk of POI [22, 26, 36–38]. However, the number of smokers in the current study population was relatively small, so the results should be interpreted with caution. The overall rate of re-operation in this study agrees with previously published results [9, 12, 39]. 

The incidence of NSD 12 months postoperatively was 33%: 15% after SJmax, 35% after SJmand, and in 40% after bimaxillary surgery. Female gender and no postoperative antibiotics significantly increased the risk of NSD. Kuhlefelt et al. identified increasing age, large mandibular displacements, manipulation of the inferior alveolar nerve, and large jaw angle as predisposing to neurosensory disturbance [40]. According to Blomqvist et al., [41] older patients are more often affected by NSD after BSSO than younger patients with similar conditions. Yoshioka et al. [42], reported gender differences for NSD (women 78% vs. men 22%) after BSSO, which the authors explained by anatomical differences in the location of the nerve canal and the hardness of the bone.

The strengths of this register-based study are related to the data registered and obtained from NROK, a national register with good geographic coverage, which increases the external validation of the results. A weakness is that NROK is a new register, launched in 2017, as it may take time to incorporate data registration as part of the clinical routine. Compliance to register data seems to be especially challenging at last follow-up visit. However, important strengths of the study are the size of the study population and registration from multiple clinics contributing to a low risk of random variation. This study is new in its kind based on data from a national orthognathic register.

Given that the NROK register is new, it would be valuable to repeat this study with the same aim and hypothesis, to compare data collected over a longer period, with more robust data, and with less dropouts.

## Conclusion

This study indicates that type of surgery, SJmand and bimaxillary surgery, significantly increased the risk of complications in terms of POI, removal of osteosynthesis material, and NSD. However, no significant difference between the type of surgery and the need for reoperation could be demonstrated.

In light of the study’s findings, the elevated risk of postoperative complications should be taken into account prior to performing BSSO procedures. Postoperative antibiotics may be considered to reduce the risk of infection. Perioperatively, it is important to handle the osteotomies and neural structure gently and ensure robust stability to reduce failure and strain on the osteosynthesis materials. Patients should be carefully informed regarding the risks of postoperative NSD. Additional factors associated with an increased risk of complications included overweight, age over forty, and smoking.

The results of this study may have clinical relevance by identifying patients at higher risk of complications associated with orthognathic surgery. Such information can assist in refining treatment planning and postoperative care to minimize risk för postoperative complications.

## Supplementary Information

Below is the link to the electronic supplementary material.

Supplementary Material 1

Supplementary Material 2

Supplementary Material 3

Supplementary Material 4

Supplementary Material 5

Supplementary Material 6

Supplementary Material 7

Supplementary Material 8

Supplementary Material 9

Supplementary Material 10

## Data Availability

No datasets were generated or analysed during the current study.
