# Supplementary material for: Factors associated with postoperative complications after orthognathic surgery – a National Swedish register-based cohort study
Source: Oral Maxillofac Surg. 2025 Dec 4;30(1):5. doi: 10.1007/s10006-025-01493-6 (PMC12675735; doi:10.1007/s10006-025-01493-6)
Supplement: Supplementary file 1 — Supplementary Material 1 [file 10006_2025_1493_MOESM1_ESM.pdf]

## Supplementary 1

Descriptive data of the study population - Patient specific characteristics

| | Total study population<br><br>n = 971 | Total population (included)<br><br>n = 428 | Total population (excluded)<br><br>n = 543 | Single jaw - maxilla (included)<br><br>n = 100 | Single jaw - maxilla (excluded)<br><br>n = 150 | Single jaw - mandible (included)<br><br>n = 130 | Single jaw - mandible (excluded)<br><br>n = 150 | Bimaxillary Surgery (included)<br><br>n = 198 | Bimaxillary Surgery (excluded)<br><br>n = 243 |
|---|---|---|---|---|---|---|---|---|---|
| **Sex** | | | | | | | | | |
| Female (ref) | 520(54%) | 241 (56%) | 279 (51%) | 52 (52%) | 71 (47%) | 85 (65%) | 91 (61%) | 104 (53%) | 117 (48%) |
| Male | 451 (46%) | 187 (44%) | 264 (49%) | 48 (48%) | 79 (53%) | 45 (35%) | 59 (39%) | 94 (47%) | 126 (52%) |
| **Age** | | | | | | | | | |
| 16-19 (ref) | 194 (20%) | 90 (21%) | 104 (19%) | 19 (19%) | 29 (19%) | 30 (23%) | 33 (22%) | 41 (21%) | 42 (17%) |
| 20-24 | 506 (52%) | 222 (52%) | 284 (52%) | 48 (48%) | 75 (50%) | 62 (48%) | 69 (46%) | 112 (57%) | 140 (58%) |
| 25-29 | 107 (11%) | 54 (13%) | 53 (9.8%) | 16 (16%) | 19 (13%) | 15 (12%) | 14 (9.3%) | 23 (12%) | 20 (8.2%) |
| 30-39 | 103 (11%) | 41 (9.6%) | 62 (11%) | 15 (15%) | 16 (11%) | 12 (9.2%) | 14 (9.3%) | 14 (7.1%) | 32 (13%) |
| 40-75 | 61 (6.3%) | 21 (4.9%) | 40 (7.4%) | 2 (2.0%) | 11 (7.3%) | 11 (8.5%) | 20 (13%) | 8 (4.0%) | 9 (3.7%) |
| **BMI** | | | | | | | | | |
| <18.5 | 37 (3.8%) | 15 (3.6%) | 22 (4.1%) | 2 (2.0%) | 3 (2.0%) | 7 (5.5%) | 7 (4.7%) | 6 (3.0%) | 12 (4.9%) |
| 18.5-25.0 (ref) | 548 (56%) | 245 (57%) | 303 (56%) | 59 (59%) | 75 (50%) | 79 (61%) | 86 (57%) | 107 (54%) | 142 (58%) |
| >25.0-30.0 | 268 (28%) | 111 (26%) | 157 (29%) | 29 (29%) | 50 (33%) | 24 (18%) | 39 (26%) | 58 (29%) | 68 (28%) |
| >30.0 | 94 (9.7%) | 41 (9.7%) | 53 (9.8%) | 8 (8.0%) | 21 (14%) | 12 (9.2%) | 15 (10%) | 21 (11%) | 17 (7.0%) |
| Data unavailable | 24 (2.5%) | 16 (3.7%) | 8 (1.5%) | 2 (2.0%) | 1 (0.7%) | 8 (6.2%) | 3 (2.0%) | 6 (3.0%) | 4 (1.6%) |
| **General disease** | 153 (16%) | 57 (13%) | 96 (18%) | 14 (14%) | 33 (22%) | 16 (12%) | 21 (14%) | 27 (14%) | 42 (17%) |
| **Smokers** | | | | | | | | | |
| Never been a smoker (ref) | 875 (90%) | 394 (92%) | 481 (89%) | 91 (91%) | 127 (85%) | 119 (92%) | 130 (87%) | 184 (93%) | 224 (92%) |
| Stopped >3 months before surgery | 29 (3.0%) | 12 (2.8%) | 17 (3.1%) | 4 (4.0%) | 4 (2.7%) | 4 (3.1%) | 6 (4.0%) | 4 (2.0%) | 7 (2.9%) |
| Stopped ≤ 3 months before surgery | 31 (3.2%) | 11 (2.6%) | 20 (3.7%) | 5 (5.0%) | 11 (7.3%) | 2 (1.5%) | 4 (2.7%) | 4 (2.0%) | 5 (2.1%) |
| Active smoker | 36 (3.7%) | 11 (2.6%) | 25 (4.6%) | 0 | 8 (5.3%) | 5 (3.8%) | 10 (6.7%) | 6 (3.0%) | 7 (2.9%) |
| **Educational level** | | | | | | | | | |
| Primary & secondary school (≤9 yr) | 232 (24%) | 99 (23%) | 133 (24%) | 24 (24%) | 33 (22%) | 32 (25%) | 41 (27%) | 43 (22%) | 59 (24%) |
| Upper secondary school (10-12 yr) | 477 (49%) | 214 (50%) | 263 (48%) | 41 (41%) | 77 (51%) | 63 (48%) | 63 (42%) | 110 (56%) | 123 (51%) |
| Higher education (ref) | 253 (26%) | 113 (26%) | 140 (26%) | 34 (34%) | 38 (25%) | 35 (27%) | 46 (31%) | 44 (22%) | 56 (23%) |
| Data unavailable | 9 (0.9%) | 2 (0.5%) | 7 (1.3%) | 1 (1.0%) | 2 (1.3%) | 0 | 0 | 1 (0.5%) | 5 (2.0%) |
| **Degree of urbanisation** | | | | | | | | | |
| Cities (≥ 50 000 inhib) | 553 (57%) | 239 (56%) | 314 (58%) | 51 (51%) | 76 (51%) | 81 (62%) | 89 (59%) | 107 (54%) | 149 (61%) |
| Towns & suburbs (≥ 5 000 inhib) | 213 (22%) | 101 (24%) | 112 (21%) | 25 (25%) | 40 (27%) | 28 (22%) | 31 (21%) | 48 (24%) | 41 (17%) |
| Rural areas (< 5 000 inhib) (ref) | 205 (21%) | 88 (20%) | 117 (22%) | 24 (24%) | 34 (23%) | 21 (16%) | 30 (20%) | 43 (22%) | 53 (22%) |

# Factors Associated with Postoperative Complications After Orthognathic Surgery – A National Swedish Register-Based Cohort Study

Oral and Maxillofacial Surgery

Carina Pekkari[1,2], Carina Kruger Weiner[1,3], Adrian Salinas Fredricson[1,2], Bodil Lund[1,4], Agneta Marcusson[5], Aron Naimi-Akbar[1,2,6]

1. Department of Dental Medicine, Division of Oral Diagnostics and Rehabilitation, Karolinska Institute, Huddinge, Sweden
2. Department of Oral and Maxillofacial Surgery, Eastmaninstitutet, Folktandvården Stockholm AB, Stockholm, Sweden
3. Department of Oral and Maxillofacial Surgery, Gävle hospital, Folktandvården, Region Gävleborg, Gävle, Sweden
4. Medical Unit for Reconstructive Plastic- and Craniofacial Surgery, Karolinska University Hospital, Stockholm, Sweden
5. Maxillofacial Unit in Linköping, and Biomedical and Clinical Sciences, Linköping University, Linköping, Sweden
6. Health Technology Assessment-Odontology (HTA-O), Faculty of Odontology, Malmö University, Malmö, Sweden

Corresponding author: carina.pekkari@ki.se
