# Supplementary material for: Factors associated with postoperative complications after orthognathic surgery – a National Swedish register-based cohort study
Source: Oral Maxillofac Surg. 2025 Dec 4;30(1):5. doi: 10.1007/s10006-025-01493-6 (PMC12675735; doi:10.1007/s10006-025-01493-6)
Supplement: Supplementary file 2 — Supplementary Material 2 [file 10006_2025_1493_MOESM2_ESM.pdf]

Supplementary 2

Descriptive data of the study population - Surgery specific characteristics

	Total study population<br>n = 971	Total population (included)<br>n = 428	Total population (exluded)<br>n = 543	Single jaw - maxilla (included)<br>n = 100	Single jaw - maxilla (excluded)<br>n = 150	Single jaw - mandible (included)<br>n = 130	Single jaw - mandible (excluded)<br>n = 150	Bimaxillary Surgery (included)<br>n = 198	Bimaxillary Surgery (excluded)<br>n = 243
**Operation time**									
<2h	290 (30%)	114 (27%)	176 (32%)	31 (31%)	65 (43%)	65 (50%)	78 (52%)	18 (9.1%)	33 (14%)
2-4h	279 (29%)	117 (27%)	162 (30%)	34 (34%)	39 (26%)	39 (30%)	44 (29%)	44 (22%)	79 (33%)
>4-9h (ref)	281 (29%)	142 (33%)	140 (26%)	28 (28%)	30 (20%)	16 (12%)	20 (13%)	98 (49%)	90 (37%)
Data unavailable	120 (12%)	55 (13%)	65 (12%)	7 (7.0%)	16 (11%)	10 (7.7%)	8 (5.3%)	38 (19%)	41 (17%)
**Antibiotic perioperative**	963 (99%)	425 (99%)	538 (99%)	100 (100%)	146 (97%)	128 (98%)	150 (100%)	197 (99%)	242 (99%)
**Antibiotic postoperative**	460 (47%)	192 (45%)	268 (49%)	41 (41%)	53 (35%)	55 (42%)	74 (49%)	96 (48%)	141 (58%)
**Osteosynthesis material**									
Osteosynthesis plates (ref)	666 (69%)	311 (73%)	355 (65%)	89 (89%)	111 (74%)	89 (68%)	100 (67%)	133 (67%)	144 (59%)
Osteosynthesis screws	175 (18%)	72 (17%)	103 (19%)	0	0	30 (23%)	36 (24%)	42 (21%)	67 (28%)
Osteosynthesis plate and screws	53 (5.5%)	23 (5.4%)	30 (5.6%)	0	0	6 (4.6%)	5 (3.3%)	17 (8.6%)	25 (10%)
Others (suture expander)	50 (5.1%)	11 (2.6%)	39 (7.2%)	11 (11%)	39 (26%)	0	0	0	0
Data unavailable	27 (2.8%)	11 (2.6%)	16 (3.0%)	0	0	5 (3.8%)	9 (6.0%)	6 (3.0%)	7 (2.9%)
**Bleeding perioperative**									
<200ml (ref)	411 (42%)	164 (38%)	247 (45%)	49 (49%)	88 (59%)	84 (65%)	95 (63%)	31 (16%)	64 (26%)
200-500ml	440 (45%)	209 (49%)	231 (43%)	44 (44%)	46 (31%)	36 (28%)	47 (31%)	129 (65%)	138 (57%)
>500ml	60 (6.2%)	26 (6.1%)	34 (6.3%)	1 (1.0%)	2 (1.3%)	2 (1.5%)	2 (1.3%)	23 (12%)	30 (12%)
Data unavailable	60 (6.2%)	29 (6.8%)	31 (5.7%)	6 (6.0%)	14 (9.3%)	8 (6.2%)	6 (4.0%)	15 (7.6%)	11 (4.5%)

Factors Associated with Postoperative Complications After Orthognathic Surgery – A National Swedish Register-Based Cohort Study

Oral and Maxillofacial Surgery

Carina Pekkari[1,2], Carina Kruger Weiner[1,3], Adrian Salinas Fredricson[1,2], Bodil Lund[1,4], Agneta Marcusson[5], Aron Naimi-Akbar[1,2,6]

1. Department of Dental Medicine, Division of Oral Diagnostics and Rehabilitation, Karolinska Institute, Huddinge, Sweden
2. Department of Oral and Maxillofacial Surgery, Eastmaninstitutet, Folktandvården Stockholm AB, Stockholm, Sweden
3. Department of Oral and Maxillofacial Surgery, Gävle hospital, Folktandvården, Region Gävleborg, Gävle, Sweden
4. Medical Unit for Reconstructive Plastic- and Craniofacial Surgery, Karolinska University Hospital, Stockholm, Sweden
5. Maxillofacial Unit in Linköping, and Biomedical and Clinical Sciences, Linköping University, Linköping, Sweden
6. Health Technology Assessment-Odontology (HTA-O), Faculty of Odontology, Malmö University, Malmö, Sweden

Corresponding author: carina.pekkari@ki.se
