# Supplementary material for: Factors associated with postoperative complications after orthognathic surgery – a National Swedish register-based cohort study
Source: Oral Maxillofac Surg. 2025 Dec 4;30(1):5. doi: 10.1007/s10006-025-01493-6 (PMC12675735; doi:10.1007/s10006-025-01493-6)
Supplement: Supplementary file 3 — Supplementary Material 3 [file 10006_2025_1493_MOESM3_ESM.pdf]

Supplementary 3

Summary of postoperative infection rate -

Analysis of single-jaw maxilla, single-jaw mandible, and bimaxillary surgery, including covariates

Postoperative infection in need of antibiotic treatment

		OR	p-value	95% CI
**Operation categories**	Single jaw – maxilla (ref)	1		(ref)
	Single jaw – mandible	3.103	0.018*	1.212–7.947
	Bimaxillary surgery	3.529	0.008*	1.393–8.943
**Gender**	Male	1.487	0.166	0.849–2.604
	Female (ref)	1		(ref)
**Age**	16–19 (ref)	1		(ref)
	20–24	1.268	0.556	0.576–2.788
	25–29	1.902	0.239	0.652–5.550
	30–39	1.361	0.610	0.416–4.455
	40–75	1.838	0.371	0.485–6.965
**BMI**	<18.5	3.119	0.082	0.865–11.25
	18.5–25.0 (ref)	1		(ref)
	>25.5–30.0	2.319	0.011*	1.216–4.423
	>30.0	1.548	0.385	0.578–4.147
**General disease**	No (ref)	1		(ref)
	Yes	1.641	0.195	0.776–3.471
**Smokers**	Never been a smoker (ref)	1		(ref)
	Stopped >3 months before surgery	1.431	0.638	0.321–6.379
	Stopped ≤ 3 months before surgery	1.369	0.700	0.277–6.759
	Active smoker	2.132	0.331	0.463–9.810
**Educational level**	Primary & secondary school (≤ 9 yr)	1.528	0.350	0.629–3.712
	Upper secondary school (10-12 yr)	1.109	0.792	0.513–2.3980
	Higher education (ref)	1		(ref)
**Degree of urbanization**	Cities (≥ 50 000 inhib)	2.484	0.025*	1.121–5.506
	Towns & suburbs (≥ 5 000 inhib)	1.276	0.611	0.499–3.261
	Rural areas (< 5 000 inhib) (ref)	1		(ref)
**Operation time**	<2 h	3.196	0.139	0.6839–14.93
	2–4 h	1.169	0.731	0.4792–2.852
	>4–9 h (ref)	1		(ref)
**Antibiotic postop**	No	2.084	0.014*	1.157–3.752
	Yes (ref)	1		(ref)
**Bleeding postop**	<200 ml (ref)	1		(ref)
	200–500 ml	2.159	0.257	0.570–8.173
	>500 ml	1.772	0.345	0.540–5.818

[*] Binary logistic regression, statistical significance $p<0.05$.

Factors Associated with Postoperative Complications After Orthognathic Surgery – A National Swedish Register-Based Cohort Study

Oral and Maxillofacial Surgery

Carina Pekkari[1,2], Carina Kruger Weiner[1,3], Adrian Salinas Fredricson[1,2], Bodil Lund[1,4], Agneta Marcusson[5], Aron Naimi-Akbar[1,2,6]

1. Department of Dental Medicine, Division of Oral Diagnostics and Rehabilitation, Karolinska Institute, Huddinge, Sweden
2. Department of Oral and Maxillofacial Surgery, Eastmaninstitutet, Folktandvården Stockholm AB, Stockholm, Sweden
3. Department of Oral and Maxillofacial Surgery, Gävle hospital, Folktandvården, Region Gävleborg, Gävle, Sweden
4. Medical Unit for Reconstructive Plastic- and Craniofacial Surgery, Karolinska University Hospital, Stockholm, Sweden
5. Maxillofacial Unit in Linköping, and Biomedical and Clinical Sciences, Linköping University, Linköping, Sweden
6. Health Technology Assessment-Odontology (HTA-O), Faculty of Odontology, Malmö University, Malmö, Sweden

Corresponding author: carina.pekkari@ki.se
