# Supplementary material for: Factors associated with postoperative complications after orthognathic surgery – a National Swedish register-based cohort study
Source: Oral Maxillofac Surg. 2025 Dec 4;30(1):5. doi: 10.1007/s10006-025-01493-6 (PMC12675735; doi:10.1007/s10006-025-01493-6)
Supplement: Supplementary file 7 — Supplementary Material 7 [file 10006_2025_1493_MOESM7_ESM.pdf]

Supplementary 7

Summary of postoperative infection rate -

Subgroups analysis of single-jaw mandible vs bimaxillary surgery, including covariates

Postoperative infection

		OR	p-value	95% CI
**Operation categories**	Single jaw – mandible	1		(ref)
	Bimaxillary surgery	1.151	0.717	0.539
**Gender**	Male	1.773	0.069	0.957–3.285
	Female (ref)	1		(ref)
**Age**	16–19 (ref)	1		(ref)
	20–24	1.249	0.610	0.531–2.939
	25–29	2.337	0.157	0.721–7.579
	30–39	1.753	0.394	0.483–6.367
	40–75	2.453	0.213	0.598–10.06
**BMI**	<18.5	4.086	0.043*	1.043–16.01
	18.5–25.0 (ref)	1		(ref)
	>25.5–30.0	2.381	0.017*	1.166–4.863
	>30.0	1.589	0.405	0.534–4.728
**General disease**	No (ref)	1		(ref)
	Yes	1.726	0.198	0.752–3.959
**Smokers**	Never been a smoker (ref)	1		(ref)
	Stopped >3 months before surgery	1.645	0.546	0.328–8.257
	Stopped ≤ 3 months before surgery	0.770	0.796	0.106–5.577
	Active smoker	2.428	0.267	0.506v11.65
**Educational level**	Primary & secondary school (≤ 9 yr)	1.547	0.383	0.580–4.125
	Upper secondary school (10–12 yr)	1.332	0.516	0.561–3.159
	Higher education (ref)	1		(ref)
**Degree of urbanization**	Cities (≥ 50 000 inhib)	2.951	0.019*	1.197–7.277
	Towns & suburbs (≥ 5 000 inhib)	1.544	0.415	0.544–4.380
	Rural areas (< 5 000 inhib) (ref)	1		(ref)
**Operation time**	<2 h	4.171	0.116	0.702–24.79
	2–4 h	1.250	0.653	0.473–3.306
	>4–9 h (ref)	1		(ref)
**Antibiotic postop**	No	2.746	0.002*	1.438–5.242
	Yes (ref)	1		(ref)
**Bleeding postop**	<200 ml (ref)	1		(ref)
	200–500 ml	2.717	0.209	0.571–12.93
	>500 ml	1.980	0.302	0.541–7.251

[*] Binary logistic regression, statistical significance $p<0.05$.

Factors Associated with Postoperative Complications After Orthognathic Surgery – A National Swedish Register-Based Cohort Study

Oral and Maxillofacial Surgery

Carina Pekkari[1,2], Carina Kruger Weiner[1,3], Adrian Salinas Fredricson[1,2], Bodil Lund[1,4], Agneta Marcusson[5], Aron Naimi-Akbar[1,2,6]

1. Department of Dental Medicine, Division of Oral Diagnostics and Rehabilitation, Karolinska Institute, Huddinge, Sweden
2. Department of Oral and Maxillofacial Surgery, Eastmaninstitutet, Folktandvården Stockholm AB, Stockholm, Sweden
3. Department of Oral and Maxillofacial Surgery, Gävle hospital, Folktandvården, Region Gävleborg, Gävle, Sweden
4. Medical Unit for Reconstructive Plastic- and Craniofacial Surgery, Karolinska University Hospital, Stockholm, Sweden
5. Maxillofacial Unit in Linköping, and Biomedical and Clinical Sciences, Linköping University, Linköping, Sweden
6. Health Technology Assessment-Odontology (HTA-O), Faculty of Odontology, Malmö University, Malmö, Sweden

Corresponding author: carina.pekkari@ki.se
