# Supplementary material for: Factors associated with postoperative complications after orthognathic surgery – a National Swedish register-based cohort study
Source: Oral Maxillofac Surg. 2025 Dec 4;30(1):5. doi: 10.1007/s10006-025-01493-6 (PMC12675735; doi:10.1007/s10006-025-01493-6)
Supplement: Supplementary file 10 — Supplementary Material 10 [file 10006_2025_1493_MOESM10_ESM.pdf]

Supplementary 10

Summary of remaining neurosensory disturbance rate -

Subgroups analysis of single-jaw mandible vs bimaxillary surgery, including covariates

**Remaining neurosensory disturbance**				
		**OR**	**p-value**	**95% CI**
**Operation categories**	Single jaw – mandible	1		(ref)
	Bimaxillary surgery	1.354	0.332	0.734–2.497
**Gender**	Male (ref)	1		(ref)
	Female	1.515	0.108	0.913–2.515
**Age**	16–19 (ref)	1		(ref)
	20–24	0.874	0.0.696	0.445–1.717
	25–29	1.865	0.213	0.700–4.968
	30–39	1.098	0.867	0.366–3.298
	40–75	2.756	0.114	0.784–9.685
**BMI**	<18.5	2.053	0.249	0.604–6.972
	18.5–25.0 (ref)	1		(ref)
	>25.5–30.0	1.122	0.709	0.613–2.052
	>30.0	1.113	0.808	0.470–2.637
**General disease**	No (ref)	1		(ref)
	Yes	1.442	0.320	0.701–2.966
**Smokers**	Never been a smoker (ref)	1		(ref)
	Stopped >3 months before surgery	2.081	0.352	0.444–9.748
	Stopped ≤ 3 months before surgery	2.207	0.415	0.328–14.82
	Active smoker	3.197	0.101	0.796–12.85
**Educational level**	Primary & secondary school (≤ 9 yr)	1.313	0.512	0.581–2.969
	Upper secondary school (10–12 yr)	1.444	0.295	0.726–2.870
	Higher education (ref)	1		(ref)
**Degree of urbanization**	Cities (≥ 50 000 inhib)	1.491	0.250	0.755–2.945
	Towns & suburbs (≥ 5 000 inhib)	1.615	0.225	0.745–3.503
	Rural areas (< 5 000 inhib) (ref)	1		(ref)
**Operation time**	<2 h	0.869	0.821	0.256–2.950
	2–4 h	0.854	0.688	0.394–1.848
	>4–9 h (ref)	1		(ref)
**Antibiotic postop**	No	1.972	0.013*	1.157–3.361
	Yes (ref)	1		(ref)
**Osteosynthesis material**	Osteosynthesis plates (ref)	1		(ref)
	Osteosynthesis screws	1.620	0.129	0.869–3.018
	Osteosynthesis plates and screws	2.083	0.127	0.811–5.352
**Bleeding postop**	<200 ml (ref)	1		(ref)
	200–500 ml	1.126	0.821	0.403–3.146
	>500 ml	1.195	0.751	0.396–3.607

* Binary logistic regression, statistical significance p<0.05.

Factors Associated with Postoperative Complications After Orthognathic Surgery – A National Swedish Register-Based Cohort Study

Oral and Maxillofacial Surgery

Carina Pekkari[1,2], Carina Kruger Weiner[1,3], Adrian Salinas Fredricson[1,2], Bodil Lund[1,4], Agneta Marcusson[5],

Aron Naimi-Akbar[1,2,6]

1. Department of Dental Medicine, Division of Oral Diagnostics and Rehabilitation, Karolinska Institute, Huddinge, Sweden
2. Department of Oral and Maxillofacial Surgery, Eastmaninstitutet, Folktandvården Stockholm AB, Stockholm, Sweden
3. Department of Oral and Maxillofacial Surgery, Gävle hospital, Folktandvården, Region Gävleborg, Gävle, Sweden
4. Medical Unit for Reconstructive Plastic- and Craniofacial Surgery, Karolinska University Hospital, Stockholm, Sweden
5. Maxillofacial Unit in Linköping, and Biomedical and Clinical Sciences, Linköping University, Linköping, Sweden
6. Health Technology Assessment-Odontology (HTA-O), Faculty of Odontology, Malmö University, Malmö, Sweden

Corresponding author: carina.pekkari@ki.se
